# Systemic cancer risk profile in neovascular age-related macular degeneration: insights into shared aging-related mechanisms from a nationwide population-based study

**DOI:** 10.18632/aging.206383

**Published:** 2026-05-22

**Authors:** Hyeong Min Kim, Yoonjong Bae, Mina Kim, Hyungwoo Lee, Hyewon Chung

**Affiliations:** 1 Department of Ophthalmology, Konkuk University College of Medicine, Seoul, Republic of Korea; 2 Department of Ophthalmology, Konkuk University Medical Center, Seoul, Republic of Korea; 3 Department of Data Science, Hanmi Pharm. Co., Ltd., Seoul, Republic of Korea

**Keywords:** neovascular age-related macular degeneration, cancer, population cohort, polygenic risk, shared susceptibility

## Abstract

Neovascular age-related macular degeneration (nAMD) is a leading cause of vision loss in older adults and is increasingly recognized as a manifestation of systemic aging involving vascular and inflammatory pathways. Emerging evidence suggests that nAMD may also be linked to systemic diseases, including malignancies. Using data from the Korean National Health Insurance Service, we conducted a nationwide, population-based cohort study of 334,091 individuals (83,742 with nAMD and 250,349 matched controls) followed for up to 10 years. Patients with nAMD showed a modest but significant increase in overall cancer risk (adjusted hazard ratio [aHR], 1.084; *P* < 0.001), with selectively elevated risks for pancreatic (aHR, 1.155; *P* < 0.001), lung (aHR, 1.128; *P* < 0.001), thyroid (aHR, 1.241; *P* < 0.001), renal (aHR, 1.177; *P* = 0.002), bladder (aHR, 1.121; *P* = 0.002), and prostate (aHR, 1.085; P < 0.001) cancers. No significant associations were observed for other malignancies. These findings indicate that nAMD may serve as a clinical marker of systemic vulnerability to selected cancers, possibly through shared angiogenic, inflammatory, and polygenic mechanisms underlying aging-related disease susceptibility.

## INTRODUCTION

Age-related macular degeneration (AMD) is one of the leading causes of irreversible vision loss among the elderly in industrialized countries, and its prevalence is projected to rise globally due to population aging. Epidemiological studies have suggested possible associations between AMD and cancer risk, but findings have been inconsistent and often did not distinguish between dry and neovascular forms [1–4]. Neovascular AMD (nAMD), characterized by choroidal neovascularization, represents the most vision-threatening form of AMD and is uniquely dependent on vascular endothelial growth factor (VEGF)-driven angiogenesis. While the ocular consequences of nAMD are well-established, mounting evidence suggests potential links between AMD and systemic diseases, including cancer [5–10]. AMD and cancer may share several common risk factors and biological mechanisms, such as advanced age, smoking, oxidative stress, chronic inflammation, and dysregulated angiogenic pathways, notably involving VEGF [1, 11–14].

Beyond angiogenesis, nAMD may also reflect broader systemic aging biology. Increasing evidence suggests that nAMD is associated with processes such as chronic low-grade inflammation (“inflammaging”), immune dysregulation, and extracellular matrix remodeling [15–17]. Cellular senescence, while an important component of aging, has been suggested to play dual roles: tumor-suppressive early via growth arrest, but tumor-promoting later through the senescence-associated secretory phenotype (SASP) [18]. In the context of AMD, several studies have demonstrated involvement of senescent retinal pigment epithelial cells and their SASP signatures [15, 16]. These mechanistic lines of evidence provide a framework in which nAMD might not only share angiogenic pathways with cancer but also intersect with systemic aging processes, potentially helping to explain its selective associations with certain malignancies. Recent genome-wide studies have revealed that both AMD and various cancer types exhibit polygenic susceptibility involving complement activation, lipid metabolism, and extracellular matrix regulation—pathways that are also implicated in tumor microenvironments and cancer progression—raising the possibility that such systemic vulnerability could extend beyond the eye.

Previous population-based studies have primarily evaluated AMD as a whole and reported mixed results, with some showing null or even inverse associations and others suggesting elevated risks for specific cancer types [2–4, 19]. However, the unique biology of nAMD and the widespread use of anti-VEGF therapies raise distinct questions about potential links between nAMD and cancer incidence, which remain incompletely addressed [2–4, 19].

We therefore carried out a nationwide cohort analysis using the Korean National Health Insurance Service (NHIS) database, aiming to examine the relationship between neovascular AMD and both overall and site-specific cancer incidence. Utilizing this large-scale, longitudinal dataset and validated diagnostic codes for both nAMD and various cancers, our study aimed to determine whether nAMD independently increases cancer risk and to identify which cancer types are most strongly linked to nAMD.

## MATERIALS AND METHODS

### Study design and population

This nationwide population-based cohort study utilized data from the Korean National Health Insurance Service (NHIS) database. The study was approved by the NHIS of Korea and the Institutional Review Board of Konkuk University Medical Center (No. KUMC IRB 2024-01-024). The NHIS database provides comprehensive nationwide health information covering more than 97% of the Korean population, including demographics, medical histories, and prescription records. All diagnoses are coded according to the International Classification of Diseases, 10th Revision (ICD-10).

Cancer cases were defined by the presence of ICD-10 codes beginning with “C” and admission history with cancer as the principal diagnosis. Additionally, cancer patients were identified through the national cancer registration program to ensure accuracy. For neovascular age-related macular degeneration, patients were identified using the special copayment reduction program code V201, which is assigned specifically for confirmed nAMD diagnoses in Korea. This registration code requires confirmation by board-certified ophthalmologists through comprehensive examination including fundus photography, optical coherence tomography, and fluorescein angiography.

The NHIS database also provides baseline demographic and comorbidity information, including body mass index, diabetes mellitus, hypertension, dyslipidemia, cardiovascular diseases, stroke, chronic kidney disease, alcohol consumption, smoking status, income status, and annual number of hospital visits. Alcohol consumption was categorized as none, mild (<15 g/day), moderate (15-29.9 g/day), and heavy (≥30 g/day). Smoking status was classified as never, former, or current smoker.

From an initial population of 1,513,912 patients aged 50 years or older who underwent NHIS retinal examinations and cancer treatments during the 10-year study period (January 1, 2010, to December 31, 2019), we excluded patients with prior cancer diagnoses (n=288,546) and those with missing data (n=761,387). The study population comprised 463,979 individuals: 83,742 patients with nAMD and 380,237 patients without nAMD. Then, 1:3 matched controls were analyzed, final 250,349 matched patients without nAMD (Figure 1).

**Figure 1:**
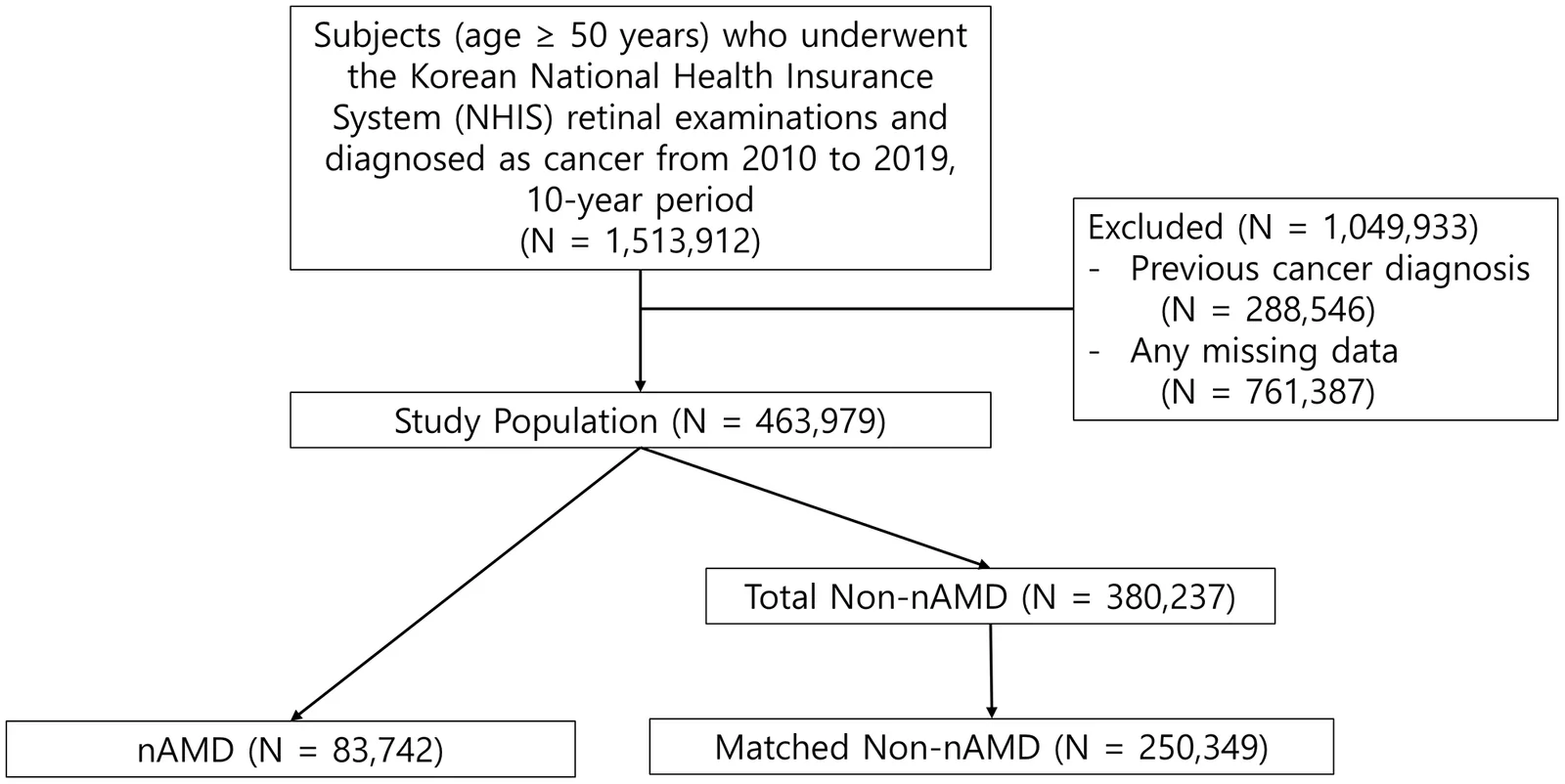
**Flow chart of the study population.** nAMD, neovascular age-related macular degeneration.

### Statistical analysis

To minimize potential selection bias and balance baseline characteristics between the groups, we performed 1:3 propensity score matching. Baseline characteristics of the matched cohort were summarized as means with standard deviations (SDs) for continuous variables and as frequencies with percentages for categorical variables. Group comparisons were performed using the Student’s *t* test for normally distributed continuous variables and the Mann–Whitney *U* test for non-normally distributed variables. Categorical variables were compared using the χ^2^ test or Fisher’s exact test, as appropriate.

Cox proportional hazards models were used to estimate hazard ratios (HRs) with 95% confidence intervals (CIs) for time-to-event outcomes. Multivariable models were adjusted for age, sex, smoking history, alcohol consumption, income status, annual number of hospital visits, and baseline comorbidities to account for potential confounding. The proportional hazards assumption was assessed using Schoenfeld residuals. Kaplan–Meier survival curves were generated to estimate cancer-free survival, and differences between groups were evaluated with the log-rank test.

Because older patients are at an increased risk of death from other age-related conditions, a competing risk analysis was additionally conducted. We utilized the Fine and Gray subdistribution hazard model, treating all-cause mortality as a competing event, to prevent the potential overestimation of cancer incidence.

All statistical analyses were two-sided. To account for the risk of Type I error associated with evaluating multiple site-specific cancers, P-values were adjusted for multiple testing using the Benjamini-Hochberg False Discovery Rate (FDR) method. An adjusted P-value < .05 was considered statistically significant. Analyses were conducted using SAS Enterprise Guide version 7.1 (SAS Institute Inc., Cary, NC, USA) and R version 4.0.1 (R Foundation for Statistical Computing, Vienna, Austria).

## RESULTS

The final analysis included 334,091 eligible individuals aged 50 years and older who underwent retinal examinations. This cohort consisted of 83,742 patients with nAMD and 250,349 matched controls without nAMD. As detailed in Table 1, the two groups were well-balanced, demonstrating no statistically significant differences in baseline demographics (age, sex, and body mass index) or major comorbidities, including diabetes, hypertension, dyslipidemia, cardiovascular diseases, and stroke, with the sole exception of chronic kidney disease. Additionally, no significant differences were observed between the groups concerning alcohol intake, smoking status, or income level.

**Table 1:** Demographics and clinical characteristics of study population.

	**nAMD** **(N = 83,742)**	**Non-nAMD** **(N=250,349)**	***P*-value**
Age, years	67.37±10.46	67.34±9.86	0.551
Gender (women)	37,287 (44.52%)	111,857 (44.68%)	0.443
BMI, kg/m^2^	24.14±3.07	24.14±3.14	0.862
Diabetes mellitus	24,553 (29.32%)	72,485 (28.95%)	0.438
Hypertension	44,809 (53.51%)	134,348 (53.66%)	0.379
Dyslipidemia	35,145 (41.97%)	104,619 (41.78%)	0.368
Heart diseases	53,661 (64.08%)	160,777 (64.22%)	0.465
Stroke	9,228 (11.02%)	27,657 (11.05%)	0.837
Chronic kidney diseases	1,728 (2.06%)	4,364 (1.18%)	**<0.001**
Alcohol consumption, g/day			
None	56,108 (67.01%)	167,746 (67.01%)	0.963
Mild (<15)	16,879 (20.15%)	50,442 (20.14%)	
Moderate (15-29.9)	6,160 (7.36%)	18,522 (7.40%)	
Heavy (≥30)	4,595 (5.48%)	13,659 (5.45%)	
Smoking status			
Never smoker	51,283 (61.24%)	153,694 (61.39%)	0.588
Former smoker	19,426 (23.20%)	57,646 (23.03%)	
Current smoker	13,033 (15.56%)	39,009 (15.58%)	
Income status			
Top third	17,855 (21.32%)	53,231 (21,26%)	0.897
Middle third	21,320 (25.46%)	63,913 (25.53%)	
Bottom third	44,567 (53.22%)	133,205 (53.21%)	

*P*-values in boldface indicate statistical significance.

nAMD, neovascular age-related macular degeneration; BMI, body mass index.

During the 10-year follow-up period, the overall incidence rate (IR) of cancer was higher among patients with nAMD compared to controls (23.55 vs. 21.01 per 1,000 person-years). Adjusted Cox proportional hazards models demonstrated that nAMD was significantly associated with an increased risk of developing any cancer (adjusted hazard ratio [aHR], 1.084; 95% CI, 1.060-1.107; *P* < .001) (Table 2).

**Table 2:** Association between neovascular age-related macular degeneration and risk of cancers.

**Cancer**		**Patients**	**IR**	**aHR**	**95% CI**	***P*-value**	***P*-value False Discovery Rate**
Any Cancer	nAMD	17,053	23.55	1.084	1.060-1.107	**<0.001**	**0.0005**
	Non-nAMD	71,028	21.01				
Stomach	nAMD	2,096	2.66	0.993	0.934-1.055	0.811	0.9531
	Non-nAMD	9,420	0.26				
Colorectal	nAMD	2,391	3.04	1.006	0.950-1.065	0.843	0.9190
	Non-nAMD	10,614	2.92				
Liver	nAMD	1,944	2.46	1.057	0.992-1.127	0.087	0.1922
	Non-nAMD	8,261	2.26				
Pancreatic	nAMD	1,544	1.95	1.155	1.074-1.244	**<0.001**	**0.0005**
	Non-nAMD	5,916	1.62				
Lung	nAMD	2,873	3.64	1.128	1.070-1.190	**<0.001**	**0.0005**
	Non-nAMD	11,158	3.06				
Thyroid	nAMD	967	1.22	1.241	1.129-1.364	**<0.001**	**0.0005**
	Non-nAMD	3,495	0.96				
Lymphoma	nAMD	321	0.40	1.053	0.927-1.196	0.423	0.6936
	Non-nAMD	1,366	0.37				
Oral	nAMD	196	0.25	1.112	0.906-1.364	0.310	0.5223
	Non-nAMD	808	0.22				
Esophagus	nAMD	220	0.28	1.021	0.882-1.182	0.781	0.9304
	Non-nAMD	983	0.27				
Gallbladder	nAMD	339	0.43	1.098	0.969-1.244	0.142	0.1802
	Non-nAMD	1,332	0.36				
Biliary	nAMD	465	0.58	0.914	0.826-1.010	0.079	0.1643
	Non-nAMD	2,269	0.62				
Laryngeal	nAMD	123	0.15	0.998	0.821-1.213	0.984	0.9531
	Non-nAMD	550	0.15				
Renal	nAMD	443	0.56	1.177	1.059-1.309	**0.002**	**0.0133**
	Non-nAMD	1,640	0.45				
Bladder	nAMD	887	1.12	1.121	1.041-1.207	**0.002**	**0.0178**
	Non-nAMD	3,476	0.95				
Nerves	nAMD	6	0.01	0.780	0.326-1.864	0.575	0.8195
	Non-nAMD	36	0.01				
Multiple myeloma	nAMD	203	0.26	1.008	0.865-1.174	0.920	0.9531
	Non-nAMD	875	0.24				
Leukemia	nAMD	197	0.25	1.058	0.904-1.237	0.483	0.8195
	Non-nAMD	830	0.23				
Skin	nAMD	741	0.93	1.060	0.977-1.149	0.161	0.4446
	Non-nAMD	3,095	0.84				
Prostate	nAMD	3,400	4.36	1.085	1.045-1.127	**<0.001**	**0.0005**
	Non-nAMD	13,817	3.82				
Testicular	nAMD	23	0.03	1.040	0.659-1.640	0.866	0.9531
	Non-nAMD	99	0.03				
Breast	nAMD	504	0.63	1.071	0.972-1.180	0.167	0.4063
	Non-nAMD	2,185	0.60				
Cervical	nAMD	117	0.15	1.038	0.849-1.269	0.719	0.9304
	Non-nAMD	530	0.14				
Corpus	nAMD	105	0.13	0.964	0.780-1.192	0.737	0.9304
	Non-nAMD	509	0.14				
Ovarian	nAMD	250	0.31	1.084	0.945-1.245	0.249	0.4446
	Non-nAMD	1,070	0.29				

*P*-values in boldface indicate statistical significance. nAMD, neovascular age-related macular degeneration; IR, Incidence Rate; CI, Confidence Interval; aHR, Adjusted Hazard Ratio.

Site-specific analyses revealed significantly elevated risks for pancreatic cancer (aHR, 1.145; 95% CI, 1.074-1.244; *P* < 0.001), lung cancer (aHR, 1.128; 95% CI, 1.070-1.190; *P* < 0.001), thyroid cancer (aHR, 1.241 95% CI, 1.129-1.364; *P* < 0.001), renal cancer (aHR, 1.177; 95% CI, 1.059-1.309; P = 0.002), bladder cancer (aHR, 1.121; 95% CI, 1.041-1.207; P = 0.002), and prostate cancer (aHR, 1.085; 95% CI, 1.045-1.127; P < 0.001). No statistically significant associations were observed for other cancer types (Table 2).

Kaplan-Meier survival analyses further supported these findings, demonstrating decreased cancer-free survival among patients with nAMD compared to controls for pancreatic, lung, thyroid, renal, bladder, and prostate cancers (Figure 2), with log-rank tests showing significant differences across these cancer types.

**Figure 2:**
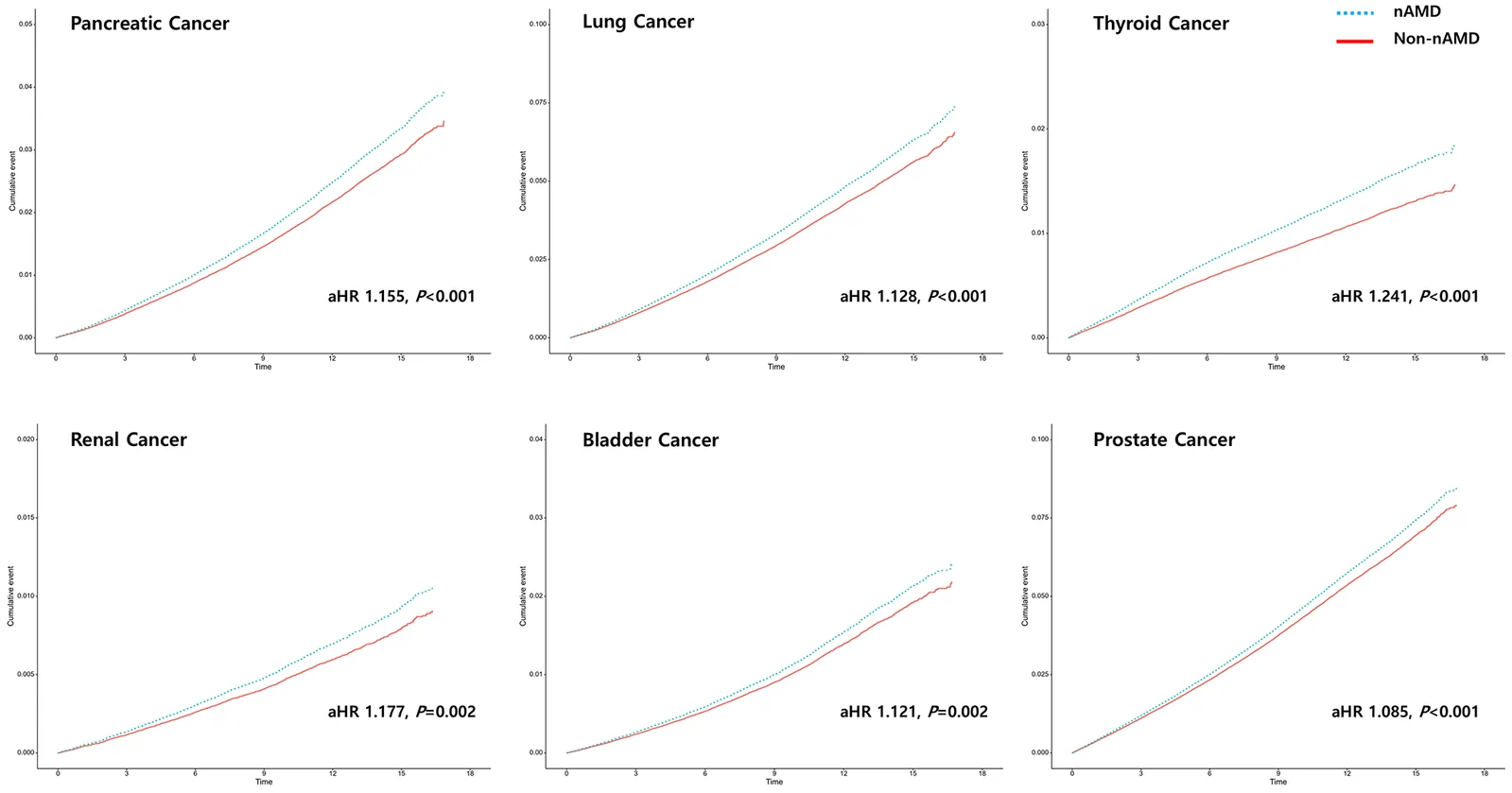
**Association between nAMD with risk of cancer by Kaplan-Meier curves.** Increased risk of pancreatic cancer (aHR 1.155, *P* < 0.001), lung cancer (aHR 1.128, *P* < 0.001), thyroid cancer (aHR 1.241, *P* < 0.001), renal cancer (aHR 1.177, *P* = 0.002), bladder cancer (aHR 1.121, *P* = 0.002), and prostate cancer (aHR 1.085, *P* < 0.001) were described.

## DISCUSSION

In this nationwide, population-based cohort study, we found that patients with nAMD had a significantly increased risk of developing cancer compared with matched controls, even after adjustment for key demographic and clinical covariates. Notably, the elevated risk was not uniform across all cancer types; it was restricted to pancreatic, lung, thyroid, renal, bladder, and prostate cancers, whereas no associations were observed for gastrointestinal, gynecologic, hematologic, or other malignancies.

### Shared angiogenic pathways between nAMD and cancer

Previous work has largely focused on angiogenesis as a common mechanism linking nAMD and cancer, as both conditions are characterized by dysregulated VEGF signaling. Indeed, the cancers that showed increased risk in our study such as lung, thyroid, renal, bladder, prostate, and pancreas, are either hypervascular tumors or show context-dependent pro-angiogenic activity. This supports the notion of shared VEGF-driven neovascularization [20–24]. A recent Korean nationwide cohort using NHIS data found that, while overall cancer risk in AMD patients was generally null, specific risks for hypervascular cancers, such as thyroid and renal cancers, were significantly increased, while risk for stomach cancer was decreased in the AMD group [4]. Our observation of increased thyroid and renal cancer risk in nAMD aligns with this literature. However, our study revealed that besides renal and thyroid cancer, bladder, prostate, lung, and pancreatic cancers are also related to the nAMD [25–30].

For bladder cancer, VEGF plays a crucial role in tumor angiogenesis, with elevated expression consistently associated with adverse clinical outcomes [31–33]. A comprehensive meta-analysis encompassing 1,285 patients from 11 studies demonstrated that tissue VEGF overexpression correlates with poor overall survival (HR 1.843, 95% CI 1.231-2.759, *P* = 0.003) and disease-free survival (HR 1.498, 95% CI 1.255-1.787, *P* < 0.001) in bladder cancer patients. These findings support the biological plausibility of shared angiogenic mechanisms between nAMD and bladder carcinogenesis [34].

In prostate cancer, histopathological analyses reveal significantly elevated microvessel density and increased VEGF expression compared to benign prostatic conditions. VEGF levels demonstrate positive correlations with advanced tumor staging, higher Gleason scores, and disease progression. Furthermore, plasma VEGF localized disease, with patients exhibiting PSA levels >20 ng/mL showing significantly higher median VEGF levels (44.5 vs. 5.5 pg/mL, *P* < 0.001). The angiogenic process in prostate cancer is driven by tumor microenvironment factors, including hypoxic conditions and androgen-mediated VEGF upregulation, which promote neovascularization essential for tumor growth and metastatic spread [35–37].

For lung cancer, VEGF overexpression is frequently observed and directly linked to enhanced angiogenesis, tumor progression, and metastatic potential. The clinical significance of anti-VEGF therapy is exemplified by bevacizumab, an anti-VEGF monoclonal antibody approved for advanced non-small cell lung cancer (NSCLC) treatment. Multiple clinical trials demonstrate that bevacizumab combined with chemotherapy significantly improves progression-free survival and overall survival in advanced NSCLC patients [38].

Pancreatic cancer presents a unique paradigm with distinct angiogenic profiles depending on histological subtype. Pancreatic ductal adenocarcinomas, comprising the majority of pancreatic cancers, are characteristically hypovascular tumors developing within a hypoxic microenvironment. The predominant pathological feature is extensive desmoplasia, dense fibrotic stroma that generates elevated interstitial fluid pressure and vascular compression, paradoxically creating a hypovascular phenotype despite VEGF expression. In contrast, pancreatic neuroendocrine tumors exhibit a hypervascular phenotype due to marked overexpression of angiogenic molecules, particularly VEGF and VEGFR, especially prominent in liver metastases. Circulating angiogenic cytokines, including VEGF and IL-8, correlate with tumor progression in pancreatic neuroendocrine patients [39–41]. The apparent contradiction in pancreatic cancer vascularity is confusing to analyze. We believe that despite the hypovascular nature of ductal adenocarcinomas, because pancreatic cancer is predominantly diagnosed at a late stage, the metastatic disease progression and tumor proliferation likely involve hypervascular angiogenic mechanisms.

An important clinical consideration is the widespread use of intravitreal anti-VEGF therapy among patients with nAMD. While these agents are administered locally, systemic absorption occurs and can transiently lower circulating VEGF levels. Theoretically, systemic VEGF suppression might be expected to reduce the risk of hypervascular malignancies. However, our observation of an increased cancer risk suggests that the systemic pro-angiogenic drive and chronic inflammatory state associated with nAMD likely override the modest systemic effects of localized ocular therapy. The elevated risk indicates that nAMD reflects a profound systemic vulnerability to pathological neovascularization that persists despite standard ophthalmic interventions.

### Beyond angiogenesis: aging, inflammation, and senescence

While angiogenesis provides a strong biological rationale, it does not fully explain why many cancers with significant angiogenic components (gastric, colorectal, hepatic, ovarian, hematologic malignancies) showed no significant relationship with nAMD. This points to a potential overlap in a broader “aging-inflammation-vasculature” axis. However, it is important to note that the hazard ratios observed in our study are modest. Therefore, rather than serving as a definitive systemic marker, nAMD may reflect a more subtle shared epidemiological susceptibility to certain aging-related processes. Cellular senescence represents one facet of aging biology and has been proposed to be tumor-suppressive early through proliferative arrest, but tumor-promoting later through SASP [18]. The selective association of nAMD with only certain cancers may thus reflect the context-dependent duality of senescence and aging protective in some tissues, tumor-promoting in others.

Several explanations are possible for the null findings. First, competing risks and lethality: fatal tumors such as gastric or hepatic cancer in Korea may obscure associations due to shortened survival. Second, screening and detection patterns: thyroid cancer may be over-detected in Korea, inflating associations, while colorectal or gastric cancers, which rely more on alternative angiogenic pathways (e.g., PlGF, PDGF, FGF, angiopoietins), may appear null [42–44]. Third, distinct tumor biology: hematologic malignancies are primarily driven by clonal hematopoiesis and marrow niche alterations, with limited reliance on VEGF-mediated angiogenesis. Moreover, breast cancer illustrates how angiogenesis mechanisms differ: intracrine VEGF signaling predominates rather than paracrine VEGF, and angiogenic activity is strongly influenced by hormone receptor status, with heterogeneity across subtypes such as triple-negative, luminal, and HER2-positive tumors [45–47]. Similarly, ovarian cancer exhibits highly variable angiogenic patterns by histological subtype, with OVA66-mediated autocrine VEGF-VEGFR2 signaling representing a distinct pathway compared to nAMD [48, 49]. Hepatocellular carcinoma also demonstrates extensive angiogenic heterogeneity and resistance to VEGF blockade, driven by hypoxia-inducible factor activation and compensatory angiogenic pathways including FGF, PDGF, and angiopoietins, explaining the lack of association with nAMD [50, 51].

Taken together, these cancer-specific examples highlight that VEGF-dependent angiogenesis alone cannot fully account for the observed selective associations. Rather, our findings underscore the importance of considering aging, inflammation, and vascular biology in an integrated framework when interpreting the relationship between nAMD and cancer risk. The absence of association with other malignancies may suggest that cellular senescence and aging exert context-dependent effects, which could be tumor-suppressive or tumor-promoting depending on tissue and biology. However, given the observational nature of our study, these interpretations remain speculative and warrant further mechanistic validation. Collectively, while the effect sizes are modest, our findings suggest a potential epidemiological link between nAMD and specific cancers that may share pathways within the aging–inflammation–vasculature axis. Rather than indicating a profound systemic vulnerability, these results highlight a subtle shared biology. Clinically, while this modest increase in risk may not currently justify the implementation of aggressive, targeted cancer screening protocols, awareness of this selective association supports the importance of comprehensive general health monitoring in nAMD patients.

### Polygenic architecture and shared genetic susceptibility

Beyond vascular and inflammatory mechanisms, emerging evidence suggests that both nAMD and several of the cancers found to be associated in this study may share overlapping polygenic architectures. nAMD is a multifactorial disorder influenced by numerous common genetic variants of modest effect, including those in the complement, lipid metabolism, and extracellular matrix pathways [52, 53]. Similarly, cancers such as pancreatic, lung, thyroid, renal, bladder, and prostate malignancies are increasingly recognized as polygenic diseases in which cumulative small-effect alleles interact with metabolic, inflammatory, and environmental factors [54]. This contrasts with malignancies driven predominantly by high-penetrance germline mutations, such as breast or ovarian cancers, where rare deleterious variants in BRCA1/2 or DNA repair genes play major causal roles [55]. These observations raise the possibility that the shared susceptibility between nAMD and selected cancers may arise from convergent networks of angiogenesis, oxidative stress, and immune dysregulation rather than from single-gene determinants. Future studies integrating genome-wide association data across AMD and cancer cohorts could help clarify whether common genetic pathways underpin this selective pattern of comorbidity.

### Study limitations and conclusion

Several limitations should be acknowledged. While we adjusted for key lifestyle factors such as smoking and alcohol consumption, the administrative nature of the NHIS database did not allow us to adjust for specific environmental exposures or individual genetic backgrounds, which may significantly influence population-specific cancer risk profiles. Misclassification of cancer diagnoses may also have occurred, although our identification of nAMD was strengthened by use of a specialized national registration code and further anchored by the requirement for anti-VEGF treatment, which makes our cohort highly reflective of real-world nAMD patients. By the same reasoning, dry AMD was not included in this analysis; while this could be considered a limitation, it also allowed us to specifically focus on nAMD with high diagnostic validity and clinical relevance. Furthermore, the NHIS database lacks detailed clinical information regarding cancer staging or severity at the time of diagnosis, precluding our ability to evaluate whether nAMD is differentially associated with early-stage versus progressive malignancies. Finally, as the study population comprises individuals within the Korean healthcare system and demographic context, results may not be directly generalizable to populations with different genetic backgrounds or healthcare structures.

In summary, our findings from this large, nationwide cohort indicate that nAMD is associated with an elevated risk of developing selected cancers, including pancreas, lung, thyroid, kidney, bladder, and prostate cancer. While shared dysregulated angiogenesis provides one biological explanation, our data also support a broader aging-inflammation-vasculature axis as a potential common ground. Clinically, these results emphasize the importance of recognizing systemic health concerns in individuals with nAMD. However, given the modest magnitude of the observed risk, these findings should be interpreted with clinical caution; they primarily underscore shared biological susceptibility rather than an immediate need for targeted cancer screening or novel preventive oncologic strategies. Future investigations should further elucidate the biological pathways underlying the observed associations and assess their implications for both ophthalmic and oncologic care. Moreover, as diagnostic coding becomes more standardized, expanding analyses to include dry AMD as well as nAMD will provide a more comprehensive understanding of the relationship between AMD as a whole and systemic cancer risk.
